# Regulation of Renal Differentiation by Trophic Factors

**DOI:** 10.3389/fphys.2018.01588

**Published:** 2018-11-12

**Authors:** Kristen Kurtzeborn, Cristina Cebrian, Satu Kuure

**Affiliations:** ^1^Helsinki Institute of Life Science, University of Helsinki, Helsinki, Finland; ^2^Medicum, University of Helsinki, Helsinki, Finland; ^3^Developmental Biology Division, Cincinnati Children’s Hospital, Cincinnati, OH, United States; ^4^GM-Unit, Laboratory Animal Centre, Helsinki Institute of Life Science, University of Helsinki, Helsinki, Finland

**Keywords:** receptor tyrosin kinase, development, kidney, intracelluar signaling, renal differentiation, morphogenesis, kidney morphogenesis

## Abstract

Classically, trophic factors are considered as proteins which support neurons in their growth, survival, and differentiation. However, most neurotrophic factors also have important functions outside of the nervous system. Especially essential renal growth and differentiation regulators are glial cell line-derived neurotrophic factor (GDNF), bone morphogenetic proteins (BMPs), and fibroblast growth factors (FGFs). Here we discuss how trophic factor-induced signaling contributes to the control of ureteric bud (UB) branching morphogenesis and to maintenance and differentiation of nephrogenic mesenchyme in embryonic kidney. The review includes recent advances in trophic factor functions during the guidance of branching morphogenesis and self-renewal versus differentiation decisions, both of which dictate the control of kidney size and nephron number. Creative utilization of current information may help better recapitulate renal differentiation *in vitro*, but it is obvious that significantly more basic knowledge is needed for development of regeneration-based renal therapies.

## Introduction

Trophic factors, also known as neurotrophic factors, have important functions outside of the nervous system (Sariola, 2001; Vega et al., 2003; Prakash et al., 2010), especially transforming growth factor beta (TGFβ) superfamily members glial cell line-derived neurotrophic factor (GDNF), bone morphogenetic proteins (BMPs), and fibroblast growth factors (FGFs), which all together with their receptors and modulators are essential for normal renal development. Other trophic factors, such as vascular endothelial growth factor (VEGF), leukemia inhibitory factor (LIF), and ephrins are less studied but also show important functions in developing functional kidneys (Weiss and Kispert, 2016).

**FIGURE 1:**
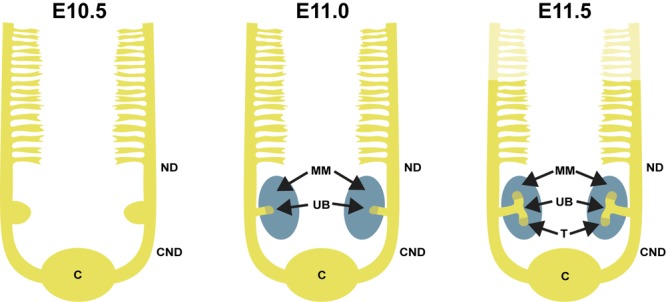
Overview of early kidney development in mouse. At embryonic day 11.0, the ureteric bud (UB) grows out from the nephric duct (ND) and enters the metanephric mesenchyme (MM). By E11.5, the UB bifurcates into two distinct UB tips (T). The cloaca is indicated as (C), and the common nephric duct is indicated as (CND).

Nephron endowment is established during the embryonic period of an individual and critically dictates renal health and function in adults; hence, a detailed understanding of the signals and events regulating renal differentiation is a necessity. Our current understanding of mammalian kidney development derives from experiments in mice, and to a lesser extent in rats and zebrafish (Davidson, 2008; Cheng et al., 2015; Drummond and Davidson, 2016). The great advances made in characterizing renal differentiation in humans during the past few years (Saraga-Babic et al., 2012; O’Brien et al., 2016; Lindstrom et al., 2018a,b,c,d; Menon et al., 2018; Wang et al., 2018) allows us to begin to ponder previous knowledge in the light of these new findings. Importantly, though these studies identified differences in rodent and human kidney development, the molecular and cellular regulation of kidney organogenesis in these species remains relatively conserved. In this review, we discuss in detail how trophic factors regulate the two major morphogenetic processes of kidney development: ureteric bud (UB) branching and nephron differentiation (Figure 1). The third important component, the stroma, is included to a significantly lesser extent. We largely focus on rodent experiments, and embryonic staging is defined for mice, if not stated otherwise. The corresponding events and timing in human fetuses were reviewed recently (Cullen-McEwen et al., 2016).

## Mouse Kidney Development – an Overview

Kidneys, like lungs, mammary and salivary glands, prostate, and seminal vesicles develop through branching morphogenesis. The characteristic growth and shape of these organs is achieved via novel branch generation that is highly specific for each organ. Thus, the pattern of epithelial branching during the differentiation of lungs is very different from that in kidneys and is largely dictated by the signals derived from the surrounding cells in the nascent mesenchymal compartment (Saxen, 1987; Lin et al., 2001).

Renal development in mammals is a multistage process during which three spatially and temporally distinct kidneys, the pronephros, mesonephros, and metanephros are formed (Figure 1). These all derive from intermediate mesoderm by spatially and temporally distinct processes (Taguchi et al., 2014; Takasato and Little, 2015). Paired nephric ducts emerge at embryonic day 8.75 (E8.75) in mice and day 28 in humans, and grow posteriorly until they reach the cloaca while inducing lateral mesonephric tubular structures (Costantini and Kopan, 2010; Little and McMahon, 2012; Combes et al., 2015). Pro- and mesonephos are transient structures that disappear by mid-gestation except in males, where the most caudal tubules of mesonephros and distal nephric duct differentiate into ductuli efferents, epididymis, vas deferens, and seminal vesicles (Jacob et al., 2012).

### Ureteric Bud Branching

The definitive kidney, the metanephros, begins forming when metanephric mesenchyme (MM) cells induce outgrowth of the UB from the nephric duct at E10.5 in mice (weeks 4–5 of gestation in humans). Subsequent renal morphogenesis is guided by reciprocal inductive interactions between the MM and the UB. After the initial UB outgrowth, it secretes signals that induce the MM to condense around the growing UB tip, forming the cap mesenchyme (CM). This is the beginning of CM-guided UB branching morphogenesis that happens rapidly until mid-gestation and slows down after E15.5 (Cebrian et al., 2004; Short et al., 2014).

**FIGURE 2:**
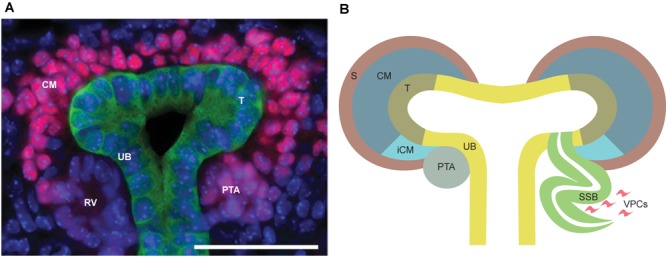
**(A)** *In vivo* representation of developing kidney at embryonic day 15.5. SIX2 staining (pink) strongly localizes to the nephron progenitors in the cap mesenchyme (CM), while being less intensive in pretubular aggregate (PTA) and almost non-existent in renal vesicle (RV). Calbindin staining (green) highlights ureteric bud (UB) epithelium. Scale bar is 50 μm. **(B)** Schematic depiction of nephrogenesis. The UB is segmented into molecularly-distinct tip (T, olive green) and stalk (UB, yellow) regions. UB tips are surrounded by CM (dark blue), including the nephron progenitors and the surrounding stroma (S, brown). A subset of the nephron progenitors in CM are induced (iCM, turquoise) to differentiate. The first differentiating nephron precursor structure is a pre-tubular aggregate (PTA, gray), which through epithelialization, turns into an S-shaped body (SSB, green) that will eventually become the functional nephron. Vascular endothelial progenitor cells (VPCs, pink) enter the cleft of the S-shaped body to begin formation of the glomerular capillary bed.

UB branching is reiterated for multiple rounds to form the entirety of the collecting duct system (Cebrian et al., 2004; Short et al., 2014). Throughout kidney development, the UB is compartmentalized into two regions, the tip and the stalk, which contain cells with distinct characteristics (Figure 2). Tip cells are the immature, proliferating cells that interact with the MM, while stalk cells, which are derivatives of tips, differentiate to form the collecting duct system (Michael and Davies, 2004; Shakya et al., 2005). The progenitor pool for the entire collecting duct system thus lies in the UB tip, and the stalk is formed by left behind tip cells. It remains unclear how specific signaling pathways control UB tip properties, but RTK signaling has been shown to keep UB cells in the tip niche (Chi et al., 2009; Kuure et al., 2010b).

### Nephron Differentiation

Nephrons are derived from a multipotent, self-renewing progenitor population of CM cells wrapping around the UB tip (Sariola, 2002; Boyle et al., 2008; Kobayashi et al., 2008). The size of the progenitor population determines the final nephron number, as indicated by progenitor depletion studies (Cebrian et al., 2014). The nephron progenitor (NP) cells rely on contacts with each other as well as with the UB cells and the interstitial progenitors to establish the progenitor niche (Figure 1), although the exact mechanisms behind niche arrangement are only beginning to be elucidated (Ihermann-Hella et al., 2018). During the active branching process, the NP population is maintained, but a subset of NP cells are also induced to differentiate and cluster to form a pretubular aggregate beneath the UB tip. The pretubular aggregate begins to epithelialize and forms the renal vesicle, which grows and is subsequently patterned into a comma-shaped and S-shaped body. The distal epithelium of the S-shaped body differentiates into the distal and connecting tubule that plumb into the collecting duct system allowing fluid to flow from the kidney. The proximal and medial segments of the S-shaped body give rise to the glomerulus and proximal tubule/Loop of Henle, respectively (Saxen and Sariola, 1987; Georgas et al., 2009). Glomerular capillary loop differentiation begins in the cleft of S-shaped body (Figure 2) simultaneously to specification of podocytes in the proximal epithelium.

After cessation of branching morphogenesis, nephrogenesis continues to produce the final number of nephrons in an individual (Cebrian et al., 2004; Short et al., 2014). In humans, nephron induction ceases *in utero* (by week 36), while in mice it lasts until post-natal day three (P3) (Cebrian et al., 2004; Hartman et al., 2007; Rumballe et al., 2011; Short et al., 2014). The mechanisms driving cessation of nephrogenesis involve loss of self-renewal in the progenitor cells that instead undergo differentiation. The molecular mechanisms driving this process are poorly understood, but recent studies identified Hamartin (encoded by *Tsc1*) as a possible regulator of progenitor aging (Volovelsky et al., 2018).

### Vascularization and Innervation

Development of the renal vasculature is not well understood, despite its critical role in renal function and overall health. Vascularization of the mouse kidney likely occurs via more than one developmental route (Abrahamson et al., 1998). Tracing of vascular development *in vivo* and studies of organ explants suggest that the main arterial network in the kidney develops independently of the glomerular vasculature. Initially, OSR1-positive precursors give rise to glomerular vascular progenitors, while at later stages in development, FLK1-positive vascular progenitors are present in the UB tip niche (Hyink et al., 1996; Mugford et al., 2008, 2009). It was also demonstrated that FLK1-positive progenitors can give rise to primitive vascular networks in cultured kidney rudiments (Tufro-McReddie et al., 1997). However, conclusive evidence of distinct developmental pathways has yet to be found.

The renal arterial tree begins forming at E12.5 in mice when several branches from the aorta enter the kidney rudiment. This network is remodeled into the single renal artery with 3–4 main lateral branches by E13.5. At E17.5, the main arterial branches extend through the medulla and terminate in the cortex where they are extensively branched to bring blood to the glomeruli (Herzlinger and Hurtado, 2014). It is not yet known which signaling factors play roles in this branching, but one possible mediator is the renin-angiotensin system which was shown to mediate vascular branching in post-natal rat kidneys (Tufro-McReddie et al., 1995).

In addition to vascularization, the functional kidney requires a neuronal network. The pattern of sympathetic innervation is closely linked to the arterial pattern in the kidney, which could indicate that the development of both networks is coordinated by the same or similar cues. Renal stroma exhibits neuronal-like cells (Sainio et al., 1994), and kidney organogenesis requires functional nerve growth factor receptor (Sariola et al., 1991). Although the regulation of the sympathetic nervous system in adults is well studied, understanding of the innervation process during renal organogenesis is largely lacking (Sariola et al., 1988).

## The Trophic Factor Signaling Pathways Essential for Renal Differentiation

### GDNF-RET Signaling

GDNF was first isolated from cultures of rat glial cell line B49 and identified as a distant member of the TGFβ superfamily with potent neurotrophic activity (Lin et al., 1993). The ability of GDNF to promote survival and differentiation of dopaminergic neurons immediately prompted further research into its use for the treatment of Parkinson’s disease. However, the blood-brain barrier prevents the use of systemic GDNF and intracranial infusion often fails to deliver sufficient GDNF to the target areas of the brain, stalling its use as a therapeutic agent for the disease (reviewed in Patel and Gill, 2007; Grondin et al., 2018). Modifications in Neurturin (NRTN), another family member expressed in developing kidney, show better spreading inside the brain (Runeberg-Roos et al., 2016).

**FIGURE 3:**
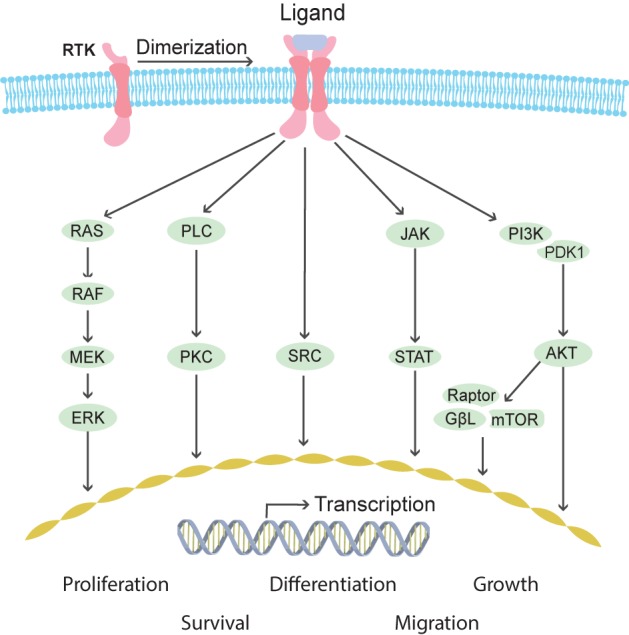
Schematic overview of pathways activated downstream of receptor tyrosine kinase (RTK) signaling. RTK receptors mediate outside signals to the interior of the cell through the cell membrane penetrating domains. Extracellular ligand binding induces receptor dimerization, which activates several intracellular cascades including MAPK, PLC/PKC, SRC, JAK/STAT, and PI3K/AKT. These signaling pathways induce transcriptional responses in the nucleus and changes of protein activities in the cytosol leading to cellular reactions such as proliferation, differentiation, growth, survival, and migration.

*Ret* proto-oncogene was characterized first in humans (Takahashi et al., 1988) and later in mice (Iwamoto et al., 1993; Pachnis et al., 1993) as an orphan tyrosine kinase receptor after which it was shortly identified as the receptor for GDNF (Durbec et al., 1996; Trupp et al., 1996). GDNF requires binding to GFRα1, a GPI-anchored cell surface co-receptor, to acquire high affinity for RET. Binding of GDNF/GFRα1 double homodimers to RET induces RET dimerization and autophosphorylation of multiple tyrosine residues. As a result, downstream signaling cascades including SRC, PLCγ, RAS-MAPK, PI3K-AKT, NFkB and JNK are activated (Figure 3; Hayashi et al., 2000; Asai et al., 2006; Davis et al., 2014). Additional complexity of intracellular signaling induction comes from alternative splicing of *Ret* resulting in three different isoforms in humans (RET9, 43, and 51) (Arighi et al., 2005).

In the shadow of its neurotrophic role, GDNF is also expressed in other organs including the developing kidney, testes, stomach, and intestine (Hellmich et al., 1996; Golden et al., 1999). The developmental role for GDNF in these organs became evident upon the generation of mice with a null mutation in GDNF; these mice showed kidney agenesis or severely hypoplastic kidneys as well as defective enteric innervation (Moore et al., 1996; Pichel et al., 1996; Sanchez et al., 1996). Indeed, mouse models with null mutations in *Ret* or *Gfrα1* demonstrated that signaling through GDNF-RET-GFRα1 is critical for kidney development, as these mice also present renal agenesis or severe renal hypoplasia (Schuchardt et al., 1994; Cacalano et al., 1998).

### FGF Signaling

The family of FGFs signal through receptor tyrosine kinase (RTK) receptors (Figure 2) to regulate organogenesis, tissue homeostasis, and metabolism via its effects on proliferation, survival, migration, and differentiation (Eswarakumar et al., 2005; Turner and Grose, 2010; Hu et al., 2013). The manifold functions in most, if not all, mammalian cell types reflect the multitude of ligands identified in mammals and alternative splicing of their receptors. A total of 22 FGF ligands, a single non-signaling receptor, and four signaling receptors have been identified to date. Of the ligands, 18 signal through FGF RTKs, of which FGFR1-3 are generally present in two major isoforms (IIIb or IIIc) (Miki et al., 1992; Chellaiah et al., 1994; Zhang et al., 2006).

Tight regulation of FGF signaling, or RTK in general, takes place at many levels, and occurs both extra- and intracellularly. It involves heparin/heparin sulfate proteoglygans (HSPG), non-TK FGFR (FGFL1), FRS2α, and Sprouty proteins (Bullock et al., 1998; Walker et al., 2016). Heparin and HSPG not only bind secreted ligands to restrict their diffusion in the extracellular matrix but also act as cofactors to provide more specificity and affinity (Rapraeger et al., 1991; Yayon et al., 1991; Matsuo and Kimura-Yoshida, 2013). Intracellular FRS2α and Sprouty largely modulate the timing, strength, and location of RAS-MAPK and PI3K-AKT cascades activation (Gotoh, 2008; Yu et al., 2011).

A multitude of FGF ligands and receptors are expressed in the developing kidney (Cancilla et al., 1999; Dudley et al., 1999). Typically, epithelial tissues, including the UB, express *Fgfr1* and -*2* IIIb splice variants while their ligands are usually expressed by the mesenchyme (Bellusci et al., 1997; Beyer et al., 2003). Conversely, *Fgfr1-2* IIIc variants localize to mesenchymal tissues and bind ligands present in the epithelium (MacArthur et al., 1995; Sun et al., 2002). Deletion of either *Frfr3* or -4 does not interfere with renal development, supporting the major signaling function for the two other receptors (Colvin et al., 1996; Weinstein et al., 1998). The function of FGFs in renal differentiation was recently reviewed (Walker et al., 2016).

### Eph/Ephrin Signaling

A bidirectional signaling module composed of 16 erythropoietin-producing hepatocellular (Eph) RTKs and nine Eph-receptor-interacting proteins (ephrins) comprises the largest RTK family in mammals (Eph-Nomenclature-Committee, 1997; Kullander and Klein, 2002). Based on sequence and function, both Ephs and ephrins are divided into A and B subfamilies with promiscuous receptor-ligand pairing within the same subfamily. A glimpse of further complexity in Eph signaling comes from the fact that both Ephs and ephrins can act as signal transducing receptors.

Activation of Eph/ephrin signaling cascades occurs exclusively upon direct contact of juxtaposed cells. Forward signaling in Eph-expressing cells triggers a large number of signal-mediating, kinase-dependent or -independent pathways, while reverse signaling in the ephrin-expressing cell causes focal adhesion kinase activation, cytoskeletal changes, and/or transcriptional responses (Binns et al., 2000; Palmer et al., 2002). The cellular responses to Eph/ephrin signaling diverge from those with RTK signaling and include cell adhesion, repulsion initiation, migration, and mitogenesis. Functionally, Eph/ephrin signaling is best recognized by several aspects of neuronal connectivity (Davy and Soriano, 2005). However, it is becoming evident that angiogenesis, craniofacial development, intestinal homeostasis, cancer, and skeletal development/homeostasis also require Eph/ephrin signaling (Bush and Soriano, 2012). Eph/ephrin signaling–mediated cell and tissue interactions in the developing kidney and urogenital system are discussed in the *Regulation of vasculature development* section.

### TGFβ and BMP Signaling

The TGFβ superfamily of secreted proteins contains at least 30 members and includes Activins, Nodals, BMPs, and Growth and Differentiation Factors (GDFs). Due to its large presentation in living organisms, it also has major functions in many diverse contexts, including developmental, homeostatic, and disease processes where members of this signaling family control basically all cell biology aspects (Weiss and Attisano, 2013). For the focus of this review, the most relevant TGFβ family members are GDNF (described earlier), BMPs, and TGFβ2.

TGFβ2, similarly, to its family members, is synthesized as a precursor and forms homodimers to activate a heteromeric receptor complex composed of type I or II transmembrane serine/threonine kinase receptors (Feng and Derynck, 2005). Regardless of the ligand-receptor complex formation mechanism, signaling activation involves constitutive type II receptor, which phosphorylates the glycine-serine domain in type I receptor to initiate RTK activity (Weiss and Attisano, 2013). Extracellular proteins, such as Noggin and Gremlin, restrict the ligand availability and thus antagonize pathway activation (Brazil et al., 2015).

The intracellular mediators, the SMAD proteins, are shared in TGFβ and BMP pathways and include both receptor-regulated SMADs (R-SMADs; SMAD2 and -3) and common mediator SMADs (co-SMAD; SMAD4). The function of SMAD2/3 is negatively regulated by inhibitory SMADs (SMAD6 and -7), which block their interaction with the receptors. Also SMAD-independent signaling, mainly through small guanosine triphosphatases (GTPase, also known as G-protein), MAPK, and PI3K pathways, may mediate TGFβ response in context-dependent situations (Lamouille and Derynck, 2011; Mu et al., 2012).

## Function of Trophic Factor Signaling in Developing Kidney

### Ureteric Bud Morphogenesis

**FIGURE 4:**
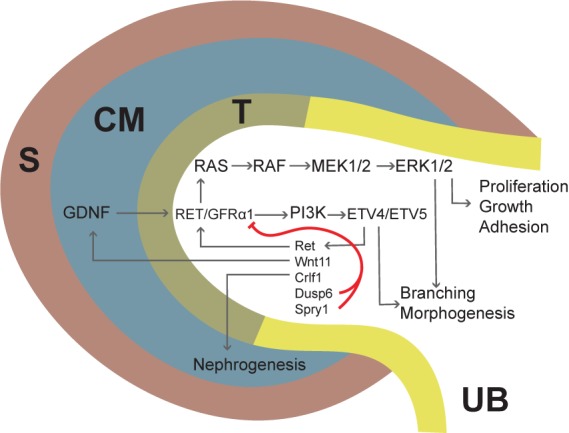
Schematic depiction of GDNF/RET/GFRα1 signaling in the ureteric bud (UB) tip during kidney development. Gdnf is first expressed in the CM (dark blue) and later also in the surrounding stroma (S, brown). GDNF binds to RET/GRFα1 complex in the UB tip (T, olive green), initiating downstream cascades such as the MAPK and PI3K pathways. Several factors are transcriptionally activated downstream of RET signaling and include Ret itself; Wnt11, which upregulates GDNF; Crlf1, which induces nephrogenesis; and Dusp6 and Spry1, which inhibit RET/GFRα1. ETV4 and -5 are transcription factors mediating the GDNF/RET response to UB epithelium and their expression depends on PI3K signaling. Signaling via the MAPK pathway is important for proliferation, growth, and adhesion, and plays a major role in branching morphogenesis (Moore et al., 1996).

*GDNF signaling* via Ret and GFRα1 is critical for kidney development and underscores the relevance of reciprocal signaling between the UB and the MM in coordinating the balance of branching and nephrogenesis (Figure 4). GDNF is expressed by the MM, and lineage tracing with a reporter line demonstrates the identity of GDNF-expressing cells as self-renewing NPs (Cebrian et al., 2014). *Ret*, on the other hand, is expressed at the tips (but not the trunk) of the UB, and *Ret*-expressing cells give rise to the entire collecting duct system (Riccio et al., 2016). *Gfrα1* mRNA is expressed both by the UB and the MM (Golden et al., 1999), however, renal organogenesis proceeds normally in a mouse model where *Gfrα1* expression is ablated in non-Ret-expressing cells (Enomoto et al., 2004), and excision of *Gfrα1* from the UB progenitors fully recapitulates the renal phenotype of the null mice (Keefe Davis et al., 2013), indicating that MM expression of GFRα1 is dispensable for kidney development.

The role of GDNF/Ret/GFRα1 signaling pathway in kidney development has been studied since the mid-nineties, but identification of its cellular functions and downstream targets remained elusive. Early *in vitro* studies suggested a mitogenic function for GDNF in UB outgrowth and branching while later experiments challenge this view by demonstrating cell organization and movement related functions (Sainio et al., 1997; Michael and Davies, 2004; Shakya et al., 2005; Chi et al., 2009; Riccio et al., 2016). Moreover, studies with a potential GDNF target *Cofilin1* (*Cfl1*) and another actin depolymerizing factor Destrin (Dstn) demonstrated that organization of epithelial cell cytoskeleton is important for new branch formation (Kuure et al., 2010a). A few genes regulated by GDNF during kidney development were identified (Pepicelli et al., 1997; Majumdar et al., 2003; Basson et al., 2005), but it wasn’t until 2009 that transcription factors *Etv4* and *Etv5* were reported as downstream targets of the pathway (Lu et al., 2009). ETV4 and ETV5 are two members of the PEA3 family of ETS transcription factors, they are expressed at the tips of the UB as well as in the MM and developing nephrons, and they functionally overlap in promoting UB morphogenesis downstream of GDNF (Lu et al., 2009). While mutations in either gene had no discernible effect on kidney development, double mutant mice presented renal agenesis and/or severe renal hypoplasia; hence Etv4 and Etv5 account for the profound effects of GDNF signaling during kidney development. There is also evidence that other transcription factors such as hepatocyte nuclear factor-1 beta (HNF1b) may contribute to signal transduction of GDNF/RET/GFRα1 and that HNF1b can directly bind to regulatory regions of Etv5 and Gfra1 to modulate transcriptional activation (Desgrange et al., 2017).

Identification of Etv4 and Etv5 as downstream targets of the GDNF/RET/GFRα1 signaling pathway opened new avenues to study the mechanisms by which GDNF exerts its trophic role on branching morphogenesis. The analysis of embryonic chimeras and Mosaic Analysis with Double Markers (MADM) mouse models with Ret and Etv4/Etv5 mutant cells demonstrated that GDNF signaling at the UB tip is required for tip cells to remain at the tip. Therefore, loss of *Ret* or *Etv4* and *Etv5* cell-autonomously compromises the ability of the tip cell to remain in the tip (Chi et al., 2009; Kuure et al., 2010b; Riccio et al., 2016). These data point toward a role of GDNF signaling in promoting cell movement and/or retention and less as a promoter of proliferation, as no differences in proliferation were detected between mutant and wild type cells (Riccio et al., 2016). These studies suggest that GDNF/RET/GFRα1 signaling may positively regulate self-renewal of UB tip-residing collecting duct progenitors. Advances in RNA sequencing techniques revealed previously unrecognized expression sites for *Gdnf*, including stromal cells (Magella et al., 2018). It remains to be seen whether future studies will identify novel functions for GDNF in processes not primarily linked to UB morphogenesis.

*FGF signaling* The earliest events in ureteric bud formation and outgrowth are well-known to depend on GDNF signaling (Costantini, 2012). However, exogenous application of FGF protein together with inhibition of TGFβ family member activin A induces supernumerary ureter budding in cultured kidneys even in the absence of GDNF/RET signaling (Maeshima et al., 2007). Thus, the FGF-induced, GDNF-independent budding may serve as a back-up mechanism that functions to ensure UB formation in the absence of GDNF/RET signaling (Basson et al., 2005; Rozen et al., 2009; Michos et al., 2010).

*Fgf10* is expressed early on in the developing kidney, and an elegant series of compound gene inactivation studies demonstrated that it is essential for UB formation in the simultaneous absence of *Gdnf* and *Spry1* (Michos et al., 2010). On their own, FGF7 and -10, secreted from the CM, regulate the extent of UB branching as shown by smaller kidneys with reduced nephron numbers in the corresponding knockout mice (Qiao et al., 1999; Ohuchi et al., 2000). A significantly more severe phenotype in UB-specific loss of *Fgfr2* than in any of the single ligand knockouts suggests that several FGF ligands converge their function on UB morphogenesis through this receptor (Zhao et al., 2004). This is further supported by the finding that UB-specific deletion of *Fgfr1*, alone or in combination with *Frs2α*, does not impact renal differentiation, while FRS2α in the UB is required for the normal branching (Sims-Lucas et al., 2009, 2011b). It appears that FGFR2 and FRS2α function both distinctly and additively in the UB lineage, challenging the view of FRS2α being the major docking protein for FGF signal transduction (Sims-Lucas et al., 2011b).

In addition to the above described cell autonomous functions, FGF signaling also critically impacts UB development via cell non-autonomous effects in the mesenchyme. Disruption of FGFR1 signaling by Pax3Cre-mediated deletion has no overt effect on renal differentiation, while a similar strategy with FGFR2 results in multiple budding, misshaped kidneys due to UB duplication, and obstructed ureters (Poladia et al., 2006; Hains et al., 2008, 2010). Simultaneous loss of both receptors allows UB outgrowth but fails to support its elongation and further differentiation, leading to renal aplasia (Poladia et al., 2006; Sims-Lucas et al., 2011a). Finally, mice lacking mesenchymal FGFR1 and concurrently deficient for FRS2α-binding in FGFR2 show remarkably milder UB defects that are also distinct from those reported for mesenchymal loss of both receptors (Poladia et al., 2006; Sims-Lucas et al., 2012). Interestingly, UB tips in the kidneys lacking mesenchymal FGFR1; FGFR2/FRS2α signaling are expanded and hyperproliferative, resembling tips seen in GDNF hypermorphic kidneys (Kumar et al., 2015). This may suggest that FGFR signaling in the MM is required to restrict biophysical and/or molecular properties of the nephron niche that reciprocally limit tip size in normal kidneys.

### Nephron Differentiation

The role of FGF signaling in the promotion of nephrogenesis became evident more than 20 years ago. Isolated MM cultures demonstrated that FGF2 can mediate the condensation and survival of nephrogenic mesenchyme while additional factors, including LIF and TGFβ2, were needed for mesenchyme-to-epithelium transformation (MET), hallmarking the major event in nephron differentiation in rats (Perantoni et al., 1995; Barasch et al., 1997, 1999; Plisov et al., 2001). The species-specific differences in MET were revealed by showing that transient activation of WNT pathway induces nephrogenesis both in mice and rats (Davies and Garrod, 1995; Kuure et al., 2007). More recent *in vitro* studies show that FGF signaling is involved in maintenance and expansion of isolated NPs (Brown et al., 2011, 2015).

*FGF signaling* has become evidently one of the major regulatory pathway in NP maintenance and differentiation. Pax3Cre-mediated simultaneous loss of *Fgfr1* and -*2* results in the failure to establish proper MM and thus, supports FGFs essential function in the formation of the initial NP pool (Poladia et al., 2006). A similar deletion strategy in the SIX2-positive population results in a remarkably less dramatic phenotype and depletion of NPs only at significantly later stages (Di Giovanni et al., 2015). The difference in phenotype severity may derive from the earlier recombination with Pax3Cre, which again suggest that FGF signaling establishes the earliest NP population or is involved in its maintenance. The essential functions of FGF signaling in creating MM and NP pool are further supported by the requirement of FGF ligand in all protocols used for stem cell-derived kidney organoid differentiation (Takasato et al., 2014, 2015; Morizane et al., 2015; Morizane and Bonventre, 2017).

Of the ligands, *Fgf7*, -*8*, -*9, -10*, and -*20* are expressed in the developing kidney and have been demonstrate to play roles in nephron differentiation (Walker et al., 2016). Genetic studies with FGF9 and -20, of which FGF9 is mainly expressed by UB epithelium and FGF20 by NPs, show that deletion of *Fgf9* alone is compatible with normal renal differentiation, while deletion of *Fgf20* causes mildly reduced kidney size and nephron number (Barak et al., 2012). Loss of all four *Fgf9;20* alleles results in renal agenesis resembling the severe phenotype seen also in MM-specific loss of both receptors (Fgfr1/2) and thus highlighting the importance of FGF signaling in establishing nephrogenic potential. On the other hand, *Fgf9 ^+^/^-^; Fgf20^-^/^-^* compound mutants show premature NP differentiation and greatly diminished total nephron number suggesting that FGF signaling activated by these ligands is needed to maintain undifferentiated status in progenitors. Also, studies with some of the FGF signaling regulators support its role in maintaining the NP population while additionally reveal control of cellular processes such survival and proliferation (Ahn et al., 2013; Motamedi et al., 2014). Interestingly, a decrease in FGF signaling coincides with cessation of nephrogenesis, suggesting a role for FGFs in increased NP cell cycle exit rates (Chen et al., 2015).

Although the majority of single ligand deletions either show normal renal organogenesis or cause embryonic lethality, deletion of *Fgf7* or *Fgf10* results in smaller kidneys with less nephrons, which likely derives from branching morphogenesis defects (Qiao et al., 1999; Ohuchi et al., 2000). However, studies with FGF8, expressed by the progenitors and differentiating renal vesicles, demonstrated that FGF8 signaling maintains NP cells but also advances nephrogenesis beyond comma-shaped body (Grieshammer et al., 2005; Perantoni et al., 2005). Molecularly, FGF8-induced signaling is required for Wnt4 and Lim1 expression in differentiating nephrons, which are severely truncated in mutant mice. The unconventional receptor FGFR-like 1 (FGFRL1), lacking the intracellular domains that provoke typical TK pathways, is also needed for *Wnt4* and *Lim1* expression and appears to regulate both UB branching and mesenchymal condensation prior to renal vesicle formation (Gerber et al., 2009). FGFRL1 binds ligands and heparin with high affinity but does not exert the mitogenic function typical for FGFR signaling (Yang et al., 2016). Rather, it promotes cellular adhesion, and some evidence suggests that it might be shed from cell membranes by a cleavage by a yet unidentified protease (Steinberg et al., 2010). The finding that intracellular domains are dispensable for its normal function supports the hypothesis that FGFRL1 could function as a secreted decoy receptor for FGF ligands, but further studies are needed to reveal its mechanistic functions in the developing kidney (Bluteau et al., 2014). Table 1 summarizes the general outcomes of genetic studies with trophic factors discussed in this review.

**Table 1:** Phenotype in genetic models of trophic factors.

Gene	Mouse model	Renal phenotype	Reference
*Gdnf*	Knockout	No kidneys, *budding*	1, 2, 3
*Gdnf*	Knockout het	Hypoplasia, *30% reduced nephron number*	4
*Gdnf*	Hypermorph	Hypodysplasia, *branching*	5
*Ret*	Knockout	70% no kidneys, 30% hypodysplasia	6
*Ret*	Ret51 hypomorph	Hypodysplasia, *branching*	7
*Gfra1*	Knockout	No kidneys, *budding*	8
*Gfra1*	UB-knockout	No kidneys, *budding*	9
*Fgf7*	Knockout	Hypoplasia, *branching*	10
*Fgf8*	Knockout	Hypodysplasia, *nephrogenesis*	11, 12
*Fgf9*	Knockout	Normal	13
*Fgf10*	Knockout	Mild hypodysplasia, *branching*	14
*Fgf20*	Knockout	Mild hypodysplasia, *nephrogenesis*	13
*Fgf9/20*	Het; knockout	Hypodysplasia; premature NP differentiation	13
*Fgf9/20*	Double knockout	Aplasia, *NP establishment*	13
*Fgfr1*	UB-knockout	Normal	15
*Fgfr2*	UB-knockout	Hypodysplasia, *branching*	15
*Frs2*	UB-knockout	Mild hypodysplasia, *branching*	16
*Frs2*	NP-knockout	Hypoplasia, cysts	17
*Fgfr1*	MM or NP-knockout	Normal	18, 19
*Fgfr2*	MM or NP-knockout	Normal	18, 19
*Fgf1/2*	MM -knockout	Aplasia, *NP maintenance*	18
*Fgfr1/2*	NP-knockout	Cystic dysplasia, NP depletion	19
*Fgfl1*	Knockout	Severe hypodysplasia, *nephrogenesis*	20
*Spry1*	Knockout	Cystic hypodysplasia, *budding & branching*	21
*Gdnf; Spry1*	Knockout	Normal, *mild branching*	21, 22
*Ret; Spry1*	Knockout	Normal	23
*Mek1*	UB-knockout	Normal	24
*Mek1*	NP-knockout	Normal	25
*Mek2*	Knockout	Normal	24, 25
*Mek1/2*	UB-knockout	Hypodysplasia, branching	24
*Mek1/2*	NP-knockout	Nephrogenesis, *NP maintenance & differentiation*	25
*Pten*	UB-knockout	Mild hypodysplasia, *branching*	26
*Shp2*	UB-knockout	Hypodysplasia, *branching*	27
*Shp2; Spry1*	UB-knockout	Hypodysplasia, *branching*	27

*Phenotype in genetic models of classical trophic factors functioning in developing kidney. Gene-column indicates the gene inactivated in a given study, Mouse model indicates the type of gene modification, and Renal phenotype first states the overall renal pathology followed by tissue of origin in italics. Abbreviations; NP, nephron progenitor; UB, ureteric bud. References: 1(Moore et al., 1996), 2(Pichel et al., 1996), 3(Sanchez et al., 1996), 4(Cullen-McEwen et al., 2001), 5(Kumar et al., 2015), 6(Schuchardt et al., 1994), 7(De Graaff et al., 2001), 8(Cacalano et al., 1998), 9(Keefe Davis et al., 2013), 10(Qiao et al., 1999), 11(Perantoni et al., 2005), 12(Grieshammer et al., 2005), 13(Barak et al., 2012), 14(Ohuchi et al., 2000), 15(Zhao et al., 2004), 16(Sims-Lucas et al., 2011b), 17(Puri et al., 2016) 18(Poladia et al., 2006), 19(Di Giovanni et al., 2015), 20(Gerber et al., 2009), 21(Basson et al., 2005), 22(Michos et al., 2010), 23(Rozen et al., 2009), 24(Ihermann-Hella et al., 2014), 25(Ihermann-Hella et al., 2018), 26(Kim and Dressler, 2007), 27(Willecke et al., 2011).*

### Vasculature Development

Similarly to the development of vasculature elsewhere in the body, the molecular regulation in the developing kidney is dictated by VEGF signaling. During the formation of the S-shaped body, vascular cells accumulate in the glomerular cleft and begin organizing into the glomerular vasculature. The beginning of the organization of the glomerular vasculature occurs concurrently with the production of VEGF from podocyte precursors, which is likely a driving factor of the glomerular vascular development as VEGF signaling from mature podocytes continues to maintain the glomerular vasculature (Eremina et al., 2003). In addition to the glomerular vasculature, VEGF derived from the renal tubule has been shown to play a role in the maintenance of peritubular vasculature (Dimke et al., 2015). Renal tubules also express TGF-β (Pelton et al., 1991; Bridgewater et al., 2011), which is required for vessel formation from endothelial progenitors (Dickson et al., 1995). It is thus likely that local cues within the cortex control arterial branching.

Vascular mural cells (VMCs) may have an important role in the arrangement of the renal vasculature. It has been shown that peritubular capillary development is mediated by angiopoietin-2, which antagonizes Ang1-dependent Tie2 signaling by the endothelia to promote VMC differentiation (Pitera et al., 2004). Vascular development is also regulated by CXCR and Eph RTK signaling (Cheng et al., 2002; Miao and Wang, 2009). Cxcl12 secreted by stromal cells and podocytes acts through its receptor Cxcr4, and mice lacking either Cxcl12 or Cxcr4 show normal kidney morphogenesis, except for a vascular patterning defect (Takabatake et al., 2009). Eph/ephrin signaling has been implicated in regulation of urorectal septation, insertion of the nephric duct into the cloaca, and glomerulogenesis (Weiss et al., 2014). EphA2 expressed in the UB epithelium shows *in vitro* negative effect on branching morphogenesis as seen by collapsing branch structures, and defects in chemotactic migration (Miao et al., 2003). It is possible that EphB4 and EphrinB2 have functions in glomerulogenesis since they are involved with angiogenesis in general (Wang et al., 1998; Weiss and Kispert, 2016), and their expression patterns suggest that they could have a role in development of non-vascular cells of glomerulus and Bowman’s capsule (Takahashi et al., 2001; Weiss and Kispert, 2016).

## Cascades Active Downstream of Trophic Factor Receptors

Several intracellular pathways are activated downstream of trophic factor-induced RTK signaling. Combinations of elegant *in vivo* and traditional *in vitro* experiments have demonstrated that RAS/MAPK, PI3K/AKT, and PLCγ cascades are essential for UB morphogenesis (De Graaff et al., 2001; Fisher et al., 2001; Tang et al., 2002; Jijiwa et al., 2004; Watanabe and Costantini, 2004; Jain et al., 2006; Willecke et al., 2011; Ihermann-Hella et al., 2014).

Important advances were made in studies aiming to maintain NPs in culture, induce isolated MM to differentiate, and generate stem cell-derived renal organoids. These efforts established the necessity of Smad, PI3K, MAPK/ERK, GSK3β, and ROCK pathways for nephrogenesis (Oxburgh and Robertson, 2002; Kuure et al., 2007; Unbekandt and Davies, 2010; Brown et al., 2013, 2015; Lindstrom et al., 2015a; Li et al., 2016; Ihermann-Hella et al., 2018), but surprisingly little efforts have been made to genetically test the requirement of individual intracellular cascades in the developing kidney (Table 1), leaving the distinct and/or synergistic functions of different pathways unknown.

### Ureteric Bud Morphogenesis

**FIGURE 5:**
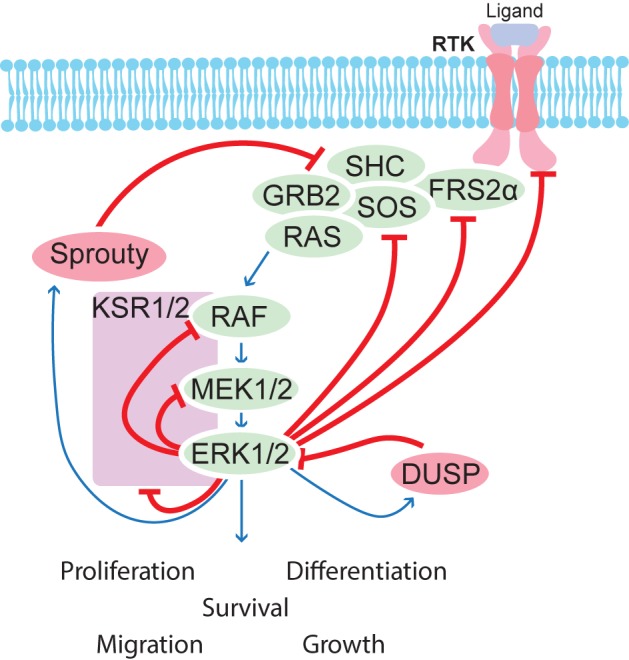
Schematic overview of feedback regulation of the MAPK pathway. Several feedback mechanisms are in place to control the MAPK pathway. ERK1/2 phosphorylates many upstream components and induces transcription of regulators such as Sproutys and DUSPs, which help to balance correct signaling strength in a given cell.

*RAS/MAPK pathway* consists of four separate cascades, extracellular signal-regulated kinases (Figure 5) (ERK1/2), Jun amino-terminal kinases (JNK1/2/3), ERK5, and p38-MAPK. Each cascade consists of three or more components including a MAPK kinase kinase (MAP3K), MAPK kinase (MAP2K), and a MAPK. Once activated, these MAP kinases activate various substrate proteins including transcription factors and protein kinases, among others (Roskoski, 2012a). MAPK signaling is controlled by positive and negative feedback loops as well crosstalk between the different pathways, and these feedback mechanisms have been reviewed elsewhere (Lake et al., 2016). Very briefly, ERK1/2 directly phosphorylates upstream components and also induces transcription of pathway inhibitors, notably here *Sprouty* and *Dusps*, which are expressed in the developing kidney (Basson et al., 2005; Lu et al., 2009).

RAS-GDP is activated by various mitogens or growth factors to become RAS-GTP which has many downstream pathways, including RAF-MEK-ERK (Pylayeva-Gupta et al., 2011; Wortzel and Seger, 2011). RAS-GTP activates RAF kinase family members (Roskoski, 2010), which in turn catalyze the activation of MEK1 and MEK2 through their phosphorylation. Activated MEK1/2 then mediate phosphorylation and activation of ERK1 and ERK2 (Roskoski, 2012b). JNK, p38, and ERK5 pathways are activated by pro-inflammatory cytokines or cellular stress (Plotnikov et al., 2011). The MEK-ERK signal transduction cascade (also known as the MAPK/ERK cascade) regulates processes such as differentiation, proliferation, transcription, metabolism, cell cycle progression, migration, survival, and adhesion while the JNK family controls apoptosis and immune cell development (Weston and Davis, 2007). While the other MAPK cascades have been extensively studied, not much is known about the MEK5-ERK5 cascade (Drew et al., 2012).

The MAPK pathway is activated in several cell types of the developing kidney (Fisher et al., 2001; Hida et al., 2002; Ihermann-Hella et al., 2014, 2018). Initially, the involvement of the MAPK pathway in UB morphogenesis was revealed by *in vitro* kidney cultures, which showed that inhibition of MEK proteins results in decreased tip cell proliferation and abnormal branching morphogenesis (Fisher et al., 2001; Watanabe and Costantini, 2004). At the UB outgrowth stage, MAPK/ERK activity is polarized on the side of the Wolffian duct, which gives rise to the bud, and is completely lost in *Ret* knockout ducts (Chi et al., 2009). Disruption of the docking site involved in MAPK and PI3K activation downstream of RET (Y1062) supports the requirement for these pathways in UB branching (De Graaff et al., 2001; Wong et al., 2005; Jain et al., 2006). However, later studies revealed that the situation is more complicated as specific mutations in distinct isoforms also have different outcomes for renal differentiation (Jijiwa et al., 2004; Jain et al., 2010). Genetic disruption of the MAPK pathway by tissue-specific deletion of *Mek1* in a *Mek2* knockout background demonstrated normal UB outgrowth but requirement for UB branch formation. Removal of MAPK activity results in elongation-only phenotype where UB fails to branch due to cell cycle progression defect and accumulation of E-cadherin on basolateral cell sides (Ihermann-Hella et al., 2014). Additional studies are needed to clarify how MAPK pathway exactly regulates E-cadherin and what are the consequences on adhesive forces in UB cells with different levels of MAPK/ERK activation.

The importance of precise regulation on MAPK activation strength and duration in developing kidneys was initially revealed by ectopic *Sprouty2* expression in the UB, which suggested changes in GDNF and FGF signaling as well as UB morphogenesis (Chi et al., 2004). Deletion of *Sprouty1* confirmed the fundamental role of negative regulation in GDNF/RET-mediated UB outgrowth (Basson et al., 2005, 2006). Moreover, the expression of not only *Sprouty1* but also two other negative regulators of the MAPK/ERK pathway, *Spred2* and *Dusp6* is induced by GDNF (Lu et al., 2009; Ola et al., 2011). Elegant genetic experiments demonstrated that synergistic GDNF/RET/Sprouty1 signaling critically balances UB outgrowth and branching (Basson et al., 2005; Rozen et al., 2009; Michos et al., 2010) while the exact roles of Spred2 and Dusp6 remain to be studied.

*PI3K/AKT pathway* The PI3Ks form a family of lipid kinases, which utilize membrane-bound phospholipids (PIP3) as secondary messengers. The class I PI3Ks mediate trophic factor-induced RTK and G protein-coupled receptor signaling, and through PIP3 recruit and activate, e.g., PI3K-dependent kinase-1 (PDK1), AKT, and small GTPases (Fruman et al., 2017). AKT is fully activated by PDK1 phosphorylation, and phosphatase and tensin homolog (PTEN) together with mammalian target of rapamycin (mTOR) complex 2 provide additional negative/positive regulation for signal transduction length, substrate selectivity, stability, and possibly subcellular localization (Manning and Toker, 2017). Activation of mTOR complex1 results in cell survival and increased protein synthesis through phosphorylation of e.g., ribosomal S6 kinase (Lamouille and Derynck, 2011). Elevated PI3K/AKT/mTOR activation is associated with many cancers, and its physiological function in normal development and homeostasis is only poorly studied. The use of pluripotent stem cells has shed new light on its function in embryonic development, self-renewal of stem cells, and maintenance of pluripotency (Yu and Cui, 2016).

Based on chemical inhibition and *in vivo* mutagenesis of *Ret* Y1062 docking site, PI3K is also required for normal UB branching (De Graaff et al., 2001; Tang et al., 2002; Jijiwa et al., 2004; Wong et al., 2005; Jain et al., 2006). The essential function of PI3K is supported by the finding that the expression of GDNF target transcription factors, *Etv4* and -*5*, requires normal PI3K activation but does not, at least at the mRNA level, depend on MAPK/ERK activity (Lu et al., 2009). FGF-stimulated, GDNF-independent UB outgrowth appears to utilize AKT activation without involvement of PI3K, and suggests differences in the use of intracellular mediators (Tee et al., 2013). Though genetic experiments specifically targeting AKT/PI3K pathway members are scarce, UB-specific deletion of *Pten*, an antagonist of PI3K activity, suggests that the UB branching pattern is shaped by PI3K activation (Kim and Dressler, 2007).

Of the other intracellular cascades activated downstream of RTK signaling, PLCγ and SRC pathways appear important for UB morphogenesis. Isoform specific mutations in tyrosine 1015 activating PLCγ causes renal abnormalities related to UB branching, but only in the context of the RET51 isoform (Jain et al., 2006). Chemical inhibition of SRC activity, on the other hand, blocks UB morphogenesis by inhibiting both new bud formation and trunk elongation while allowing abundant nephrogenesis to take place (Kuure et al., 2007). Some indications of p38 MAPK functions in collecting duct differentiation were suggested by the studies with integrin-linked kinase in the UB (Smeeton et al., 2010), but its fundamental role remains to be studied. In conclusion, combined approaches of biochemistry, cell biology, and genetics will be needed to better understand the function of individual intracellular cascades and even more important, to interpret how they cooperate in a context-dependent manner to mediate extracellular stimuli of trophic factors.

### Nephrogenesis

*SMAD pathway* Without activated TGFβ superfamily signaling, the SMAD proteins, the intracellular mediators of this pathway, are shuttling constantly between the cytoplasm and nucleus (Xu et al., 2002). Upon the tertiary ligand-receptor complex formation, the receptor-regulated SMAD proteins specific to TGFβ, R-SMAD2/3, and those activated downstream of BMP, R-SMAD1/5/8, are recruited to the complex. This triggers their phosphorylation by type I receptor and again frees them into the cytosol, where they are able to form heterodimeric complexes with common SMAD4. The trimeric complex of two R-SMADs together with SMAD4 then accumulates in the cytosol from where it is imported into the nucleus (Hill, 2009). Additional input from the other upstream pathways is important in determining the duration, strength, and response of the signal (Massague, 2012).

Several SMAD proteins localize to NPs while being downregulated in the induced nephron precursors (Oxburgh and Robertson, 2002). This suggests that BMP/TGFβ-induced SMAD signaling exhibits essential functions in NP biology and the importance of BMP7 signaling was demonstrated in classical knockout studies (Dudley et al., 1995; Luo et al., 1995). More recent experiments reveal that BMP7-induced signaling plays an important role in progenitor proliferation, survival, and preventing premature differentiation (Blank et al., 2009; Tomita et al., 2013). Mitogenic response appears to be mediated via Jun (also known as c-jun) N-terminal kinases (JNKs) while signaling through the SMAD pathway promotes progenitor differentiation via commitment to transient amplifying cells (Brown et al., 2013). Accordingly, SMAD inhibition maintains progenitors in a more stem-like state, as shown by the expansion of the CITED1/SIX2-positive compartment (Brown et al., 2015), which is considered as the least differentiated NP population in the developing kidney (Costantini and Kopan, 2010). Long-term *in vitro* culture of NPs supports the requirement for BMP and Rho kinase pathways as the addition of BMP7 and ROCK inhibitor together with WNT activation and FGF supply enables their maintenance (Unbekandt and Davies, 2010; Li et al., 2016).

*RTK activated intracellular pathways* WNT/β-catenin signaling is the major driver that pushes NPs to differentiate (Stark et al., 1994; Kuure et al., 2007; Park et al., 2007). Additionally, the JNK pathway appears to be activated downstream of WNT-induced nephron differentiation, at least in cultured colony-forming progenitors (Osafune et al., 2006). The complexity in control of the self-renewal versus differentiation decision was demonstrated by a series of time-lapse imaging of nephrogenesis in *in vitro* cultured kidneys (Lindstrom et al., 2015a,b). It showed that balancing signaling strengths, as shown for PI3K activation, determines whether to maintain a stem-like character or converge the differentiation program. Similarly, our recent study showed that MAPK/ERK activation, as revealed by live-imaging of embryonic kidneys isolated from Förster resonance energy transfer (FRET) biosensor of ERK, is heterogeneous among the NP population and very strong in RVs (Ihermann-Hella et al., 2018). We showed by NP-specific inactivation that MAPK/ERK activity controls niche organization and communication with extracellular matrix and is essential for normal NP differentiation. In the absence of MAPK activity, nephrogenesis proceeded quite normally up to RV stage but then halted almost exclusively. Interestingly, although strong ERK activation and pERK1/2 signal is detected in the connecting piece of nephron to collecting duct, no obvious defects were seen in this process. It remains to be studied how activation strength in trophic factor-induced intracellular pathways contributes to the biophysical properties of progenitor niche and nephron differentiation, e.g., in specifying nephron segments.

## Future Aspects

Since the identification of the first trophic factors in the 1970s, remarkable progress has been made toward identifying additional members of this large group of signaling molecules and understanding the varied mechanisms by which they exert their trophic roles. These mechanisms often present redundancy and/or synergism in a tissue- and time-dependent manner. Two somewhat overlapping lines of research are now providing a stepping stone to better understand the role of these trophic factors in kidney development. On the one hand, there has been a significant effort from the McMahon lab and others to characterize human kidney development not only at an anatomical level but also at the molecular level using single-cell sequencing (Lindstrom et al., 2018a,b,c,d; Menon et al., 2018; Wang et al., 2018). Complementary to these studies, and somehow preceding them, researchers can now induce differentiation of human embryonic stem cells into kidney organoids that, among many other applications, can be used to finely characterize signaling pathways that drive human kidney organogenesis (Lam et al., 2014; Taguchi et al., 2014; Takasato et al., 2014; Morizane et al., 2015). As is often the case with basic research, answering some questions will also open many new lines of inquiry, and there is still much to learn about how these growth factors drive and modulate the delicate process of kidney organogenesis.

## Author Contributions

All authors contributed equally to the writing of this review manuscript.

## Conflict of Interest Statement

The authors declare that the research was conducted in the absence of any commercial or financial relationships that could be construed as a potential conflict of interest.
